# Activation of the GLP-1 Receptors in the Nucleus of the Solitary Tract Reduces Food Reward Behavior and Targets the Mesolimbic System

**DOI:** 10.1371/journal.pone.0119034

**Published:** 2015-03-20

**Authors:** Jennifer E. Richard, Rozita H. Anderberg, Andreas Göteson, Fiona M. Gribble, Frank Reimann, Karolina P. Skibicka

**Affiliations:** 1 Department of Physiology, Institute of Neuroscience and Physiology, The Sahlgrenska Academy at the University of Gothenburg, Gothenburg, Sweden; 2 MRC Metabolic Diseases Unit and Institute of Metabolic Science, University of Cambridge, Cambridge, United Kingdom; Hosptial Infantil Universitario Niño Jesús, CIBEROBN, SPAIN

## Abstract

The gut/brain peptide, glucagon like peptide 1 (GLP-1), suppresses food intake by acting on receptors located in key energy balance regulating CNS areas, the hypothalamus or the hindbrain. Moreover, GLP-1 can reduce reward derived from food and motivation to obtain food by acting on its mesolimbic receptors. Together these data suggest a neuroanatomical segregation between homeostatic and reward effects of GLP-1. Here we aim to challenge this view and hypothesize that GLP-1 can regulate food reward behavior by acting directly on the hindbrain, the nucleus of the solitary tract (NTS), GLP-1 receptors (GLP-1R). Using two models of food reward, sucrose progressive ratio operant conditioning and conditioned place preference for food in rats, we show that intra-NTS microinjections of GLP-1 or Exendin-4, a stable analogue of GLP-1, inhibit food reward behavior. When the rats were given a choice between palatable food and chow, intra-NTS Exendin-4 treatment preferentially reduced intake of palatable food but not chow. However, chow intake and body weight were reduced by the NTS GLP-1R activation if chow was offered alone. The NTS GLP-1 activation did not alter general locomotor activity and did not induce nausea, measured by PICA. We further show that GLP-1 fibers are in close apposition to the NTS noradrenergic neurons, which were previously shown to provide a monosynaptic connection between the NTS and the mesolimbic system. Central GLP-1R activation also increased NTS expression of dopamine-β-hydroxylase, a key enzyme in noradrenaline synthesis, indicating a biological link between these two systems. Moreover, NTS GLP-1R activation altered the expression of dopamine-related genes in the ventral tegmental area. These data reveal a food reward-suppressing role of the NTS GLP-1R and indicate that the neurobiological targets underlying food reward control are not limited to the mesolimbic system, instead they are distributed throughout the CNS.

## Introduction

Feeding behavior is thought to be regulated by two intermingled central nervous system (CNS) pathways: homeostatic and hedonic [1–3]. Hedonic eating is suggested to be one of the main culprits behind the increasing rates of obesity [4]; it is therefore incredibly important to investigate the mechanisms underlying the drive to excessively consume palatable food. Dopamine within the mesolimbic system is considered crucial for the hedonic aspects of feeding behavior [5]. The intake of palatable food increases the activity of dopamine neurons in the ventral tegmental area (VTA) and the release of dopamine in the nucleus accumbens [6,7]. These two mesolimbic nuclei are therefore considered crucial for food reward control and remain the main neuroanatomical sites investigated in studies with a food reward focus. Here, however, we suggest that this may be a limiting perspective and propose that the focus of the food reward regulation studies should be expanded; we suggest that the nucleus of the solitary tract (NTS) in the hindbrain may be an important neural substrate for regulation of food reward.

Glucagon-like peptide-1 (GLP-1) is one of the key signals involved in the CNS regulation of feeding behavior [8–11]. GLP-1 is produced in intestinal L-cells and in the hindbrain, primarily in the NTS [12]. GLP-1-producing neurons innervate the classic homeostatic energy balance-controlling brain regions that include the hypothalamus and the NTS [12–14] and GLP-1 receptors (GLP-1R) have been found in these areas [15]. Hypothalamic and hindbrain GLP-1R have been shown to play an important role in the homeostatic regulation of food intake [16,17]. Stimulation and blockade of these receptors reduces and increases the amount of food eaten respectively [16,17]. However, we have recently demonstrated that in addition to reducing the amount of food eaten central GLP-1R activation also reduces food reward behavior which may represent the hedonic aspect of feeding behavior [18]. This impact of GLP-1R on food reward behavior is thought to be mediated through its actions on the mesolimbic GLP-1R, in areas commonly associated with reward, such as the nucleus accumbens and the VTA [18]. Collectively these findings fit with the view that homeostatic aspects of feeding are regulated by the action of GLP-1 in classically homeostatic areas and reward-driven aspects are controlled by the mesolimbic actions of GLP-1. Here we want to challenge this view by hypothesizing that GLP-1 can impact food reward by acting on its receptors in the hindbrain’s NTS. Our hypothesis is strengthened by two recent reports that show that the fat produced hormone leptin and the hypothalamic neuropeptide orexin impact on food reward behavior by acting on their NTS receptors [19,20].

To test the role of NTS GLP-1R stimulation in food-motivated behavior and food reward we utilized two behavioral models typically used to determine the addictive properties of substances, the progressive ratio operant conditioning test and conditioned place preference (CPP) paradigm. The first task is a well-established test of motivated behavior [21], where the higher the motivation to obtain the rewarding substance the harder the rat is willing to work (press a lever) for it. In the conditioned place preference test the more rewarding the animal finds the food, the more time it will spend in the food-associated compartment based on a learned association between the food and the visuospatial cues from the reinforced location. This procedure allows for testing in the absence of food, reducing any potential confounding effects of postingestive feedback during testing. NTS noradrenergic neurons and neurons containing cholecystokinin (CCK) send ascending projections from the NTS to the nucleus accumbens and the VTA providing a possible pathway in which GLP-1 acting within the brainstem can affect the reward system [22]. Therefore in order to determine a potential link between GLP-1 activation with activation of these two neuronal phenotypes, we first evaluated whether central GLP-1R activation changes the gene expression of NTS CCK, and also two enzymes key for noradrenaline production tyrosine hydroxylase (TH) and dopamine-β-hydroxylase (dBH). We next utilized a transgenic mouse [14] that expresses yellow fluorescent protein (YFP) selectively in the GLP-1-producing neurons to determine whether these neurons innervate TH-positive neurons in the NTS allowing a monosynaptic connection between GLP-1R activation and the mesolimbic system. To further assess the impact of NTS GLP-1R activation on the mesolimbic function we also determined the gene expression of key reward behavior associated genes in the VTA and nucleus accumbens induced by NTS GLP-1R stimulation. Collectively our findings support the notion that the caudal brainstem participates in the control of food reward and food-motivated behavior and identify potential mechanisms via which the NTS GLP-1R-evoked signal may be transmitted to the mesolimbic pathways to reduce food-reward behavior.

## Materials and Methods

### Animals

Male Sprague-Dawley rats (180–250 g at arrival and 400 g during the drug administration tests, Charles River, Germany) were housed in a 12 h light/dark cycle, in individual cages with free access to chow and water, except during the period of operant testing or peanut butter consumption. Adult female and male mGLU-124 Venus yellow fluorescent protein transgenic mice (YFP-PPG mice; University of Cambridge, United Kingdom [23]) were housed in plastic cages with water and standard chow available ad libitum. All studies were carried out with ethical permissions from the Animal Welfare Committee of the Göteborg University, in accordance with legal requirements of the European Community (Decree 86/609/EEC). All efforts were made to minimize suffering.

### Surgery

**Fig 1:**
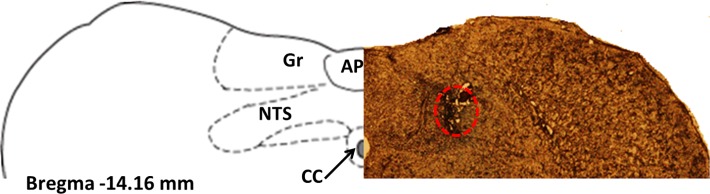
Representative photomicrograph of a coronal section of the rat brain at the level of the NTS illustrating the microinjection site (encircled area) for the behavioral experiments (right panel) and a schematic representation of the NTS from the rat atlas (Paxinos and Watson, 1998) (left panel). Area postrema (AP), central canal (cc), gracile nucleus (Gr).

Rats were implanted with a guide cannula targeting the NTS or the lateral ventricle (26 gauge; Plastics One, Roanoke, VA) under ketamine anesthesia as described previously [24,25]. The following coordinates were chosen for the NTS: ±0.75mm from the midline/on occipital suture/-4.9mm, with injector aimed 6.9 mm ventral to skull; the following coordinates were used for the lateral: ±1.6 from the midline, 0.9 mm posterior to bregma, and 2.0 mm ventral to skull, with injector aimed 4.0 mm ventral to the skull. NTS cannula placement was first verified one week after the surgery by the measurement of the sympathoadrenal-mediated glycemic response by microinjection of 5-thio-D-glucose (24μg/0.3μl) [26]. An elevation of plasma glucose levels by 100% or more was required for subject inclusion in the study. Placement was also assessed histologically *post mortem* by injection of India ink (0.3 μl volume matched drug delivery in the experiments). Only rats whose dye injection site was found within the NTS were included in the data analysis (Fig. 1). The lateral ventricle placement was verified with the angiotensin II drinking test. Angiotensin II was injected at a dose of 20ng in 2μL of aCSF and water intake was measured immediately. Rats that drank at least 5ml of water in 30 min, were considered to have a correct cannula placement.

### Drugs

Exendin-4 (Ex4), GLP-1 (7–36) and angiotensin II, were purchased from Tocris (Bristol, UK) dissolved in aCSF (vehicle for all central injections) and stored as aliquots in -20°C.

### Palatable food-choice test

Preference for palatable food (peanut butter) or chow was determined by offering the two foods simultaneously on the test day. In order to determine the effect of NTS GLP-1R activation on the palatable food choice, food presentation was preceded by intra-NTS microinjection of Ex4 (0.05 μg) or aCSF and the amount of chow and peanut butter eaten was measured at 1,3, and 6h. Injections were done in a counterbalanced, Latin square design with at least 48h separating each injection condition. Variations in consumption due to neophobia were reduced by familiarizing all the rats with the peanut butter on at least one occasion prior to the test day. The amount of calories consumed was computed using the following values: 4.1 kcal/g for chow and 6.6 kcal/g for peanut butter.

### PICA test

To determine whether Ex4 injections into the NTS are associated with nausea, the PICA response was measured (consumption of non-nutritive substances that mimics emesis in species not capable of the emetic response). Rats were allowed to sample kaolin for at least 3 days before the Ex4 injection to avoid association of kaolin with Ex4 injections. Kaolin intake and chow intake were measured at 1,3,6, and 24h after injection in rats mildly food-restricted overnight (10g of chow available overnight). The dose of Ex4 (0.05 μg) was chosen to reduce chow intake by at least 50%.

### Operant conditioning

Food-induced operant conditioning training and testing were conducted in rat conditioning chambers (Med-Associates, Georgia, VT, USA) as described previously [18,27]. Rats were trained to press a lever for a 45mg sucrose reward. Training was conducted in four stages: rats were first trained on the fixed ratio 1 (FR1) schedule (single press on the active lever resulted in the delivery of one sucrose pellet), followed by FR3 and FR5 (3 and 5 presses per pellet respectively), where a minimum of 50 responses per session on the active lever was required for the advancement to the next schedule, culminating with progressive ratio conditioning until stable responding was achieved. Each progressive ratio session lasted for 90 min. Responding was considered stable when the number of pellets earned per session did not differ more than 15% for three consecutive sessions. All operant response testing was performed after the responses stabilized. Rats received drug injections early in the light cycle after partial (10g of chow available) overnight food restriction. This paradigm was chosen to provide a higher motivation to work for sucrose, which was hypothesized to be subsequently attenuated by Ex4 or GLP-1. Injections were done in a counterbalanced, Latin square design with at least 48h separating each injection condition. Testing was performed during the light cycle. Immediately after the operant boxes test rats were returned to their home cages and offered chow, the amount of chow eaten was measured after 1h and 22h. Body weight was measured at 22h. Locomotor activity was measured during the operant testing with infrared beams installed inside the operant chambers.

### Conditioned place preference (CPP)

The CPP test was performed in rats using an apparatus that comprised of two connected chambers with distinct visual and tactile qualities (Med-Associates). Initial preference for one chamber was assessed on the first and second day with 20 min pretests on each day, and the least preferred compartment was determined by taking the average amount of time spent in each compartment across the two pretest days. Subsequently the least preferred compartment was paired with 4g of palatable food (chocolate or peanut butter). The pretest was followed by 16 days of conditioning sessions (2 sessions per day). One day following the last conditioning session rats were injected (intra-NTS) with vehicle (aCSF) or Ex4 (0.05μg) 30min before being placed in the CPP apparatus for 10 min. The behavior of the animals was detected by infrared beams in each chamber and time spent in each compartment was determined. To assure that all palatable food was always consumed during the training sessions the rats had restricted access to chow in their home-cages (to 70% of normal daily chow intake) throughout the CPP experiment.

### GLP-1 fiber detection

Mice were anaesthetized with ketamine/xylazine solution and perfused transcardially with heparinized saline followed by fresh fixative solution (paraformaldehyde (PFA, 4%) in 0.1 M phosphate buffer). The brains were collected, and cut into coronal 25 μm sections using a cryostat, sections were then collected into tubes containing tissue storage solution consisting of 50 ml glycerin, 50ml ethylene glycol and 100 ml 0.1 M phosphate buffer (pH 7.5) and stored until use in 4°C. The sections were washed (3 x 15 min) in TNT with Triton-X (0,1%) (Sigma-Aldrich St.Louis, MO, USA). For **tyrosine hydroxylase (TH) visualization**, the sections were incubated for two days in TNB blocking solution (Perkin Elmer, Akron, Ohio, USA) with 1:2000 Goat polyclonal antibody to TH (ab6211, Abcam, Cambridge, UK). The sections were then washed in TNT with Triton-X (0,1%) and incubated in TNB blocking solution with 1:1000 Donkey anti-goat Alexa Fluor 568 (ab36001, Abcam). The cell nuclei were stained with DAPI (1:5000; Life Technologies, Carlsbad, CA, USA). The sections were then washed in TNT (2 x 15 min), submerged in 0.1M PB and mounted on microscope slides (Superfrost Plus, Menzel) together with ProLong Gold Antifade (Life Technologies). The GLP-1 fibers were visualized with a confocal microscope (LSM 700; Carl Zeiss, Oberkochen, Germany).

### RNA isolation and mRNA expression

VTA and nucleus accumbens gene expression levels were measured after intra-NTS injection of Ex4 (0.05μg) or vehicle (aCSF). The following genes were examined: *TH*, *Drd1a*, *Drd2*, *Drd3*, *Drd5*, *Slc6a3*, *Gad1*, *Creb1*, *FosB*. They were selected because of their previously reported role in reward behavior regulation or their connection to GLP-1. Ninety minutes after Ex4 or aCSF injection the brains were rapidly removed and the VTA and nucleus accumbens were dissected using a brain matrix, frozen in liquid nitrogen and stored at -80°C. Individual brain samples were homogenized in Qiazol (Qiagen, Hilden, Germany) using a TissueLyzer (Qiagen). Total RNA was extracted using RNeasy Lipid Tissue Mini Kit (Qiagen) with additional DNAse treatment (Qiagen). RNA quality and quantity were assessed by spectrophotometric measurements (Nanodrop 1000, NanoDrop Technologies, USA). For cDNA synthesis iScript cDNA Synthesis kit (BioRad) was used. Real-time RT PCR was performed using TaqMan probe and primer sets for target genes chosen from an on-line catalogue (Applied Biosystems; reference numbers were as follows: Actb-Rn00667869_m1, TH-Rn00562500_m1, Drd1a-rCG24308, Drd2-rCG57985, Drd3-rCG52650, Drd5-rCG35929, Slc6a3-rCG41956, Gad1-rCG26162, Creb1-rCG22512, Fos-rCG20898). Gene expression values were calculated based on the ^ΔΔ^ *C*_t_ method [28], where the vehicle-injected group was designated as the calibrator. Beta-actin was used as a reference gene. To determine the NTS gene expression of CCK (Rn00563215_m1) and two enzymes key to noradrenaline synthesis, dBH (Rn00565819_m1) and TH, rats were injected into the lateral ventricle with 0.2 μg of Ex4 or vehicle (aCSF) and ninety minutes later an NTS-enriched dorsal brainstem tissue block was dissected. The tissue was rapidly frozen, stored at -80°C, and mRNA extraction, cDNA synthesis and TaqMan PCR were performed as described above. Peptidylprolyl isomerase A (ppia, Rn00690933_m1) was determined to be a more stable gene for the NTS than β-actin, and used as a control gene.

### Statistical analysis

All the data are presented as mean ± Standard Error of the Mean (SEM). Statistical significance was analyzed using Student’s t test, one- or two-way ANOVA when appropriate (GraphPad Software, Inc., San Diego, CA). P- values lower than 0.05 were considered statistically significant.

## Results

### NTS GLP-1R activation preferentially affects intake of palatable food but not chow

**Fig 2:**
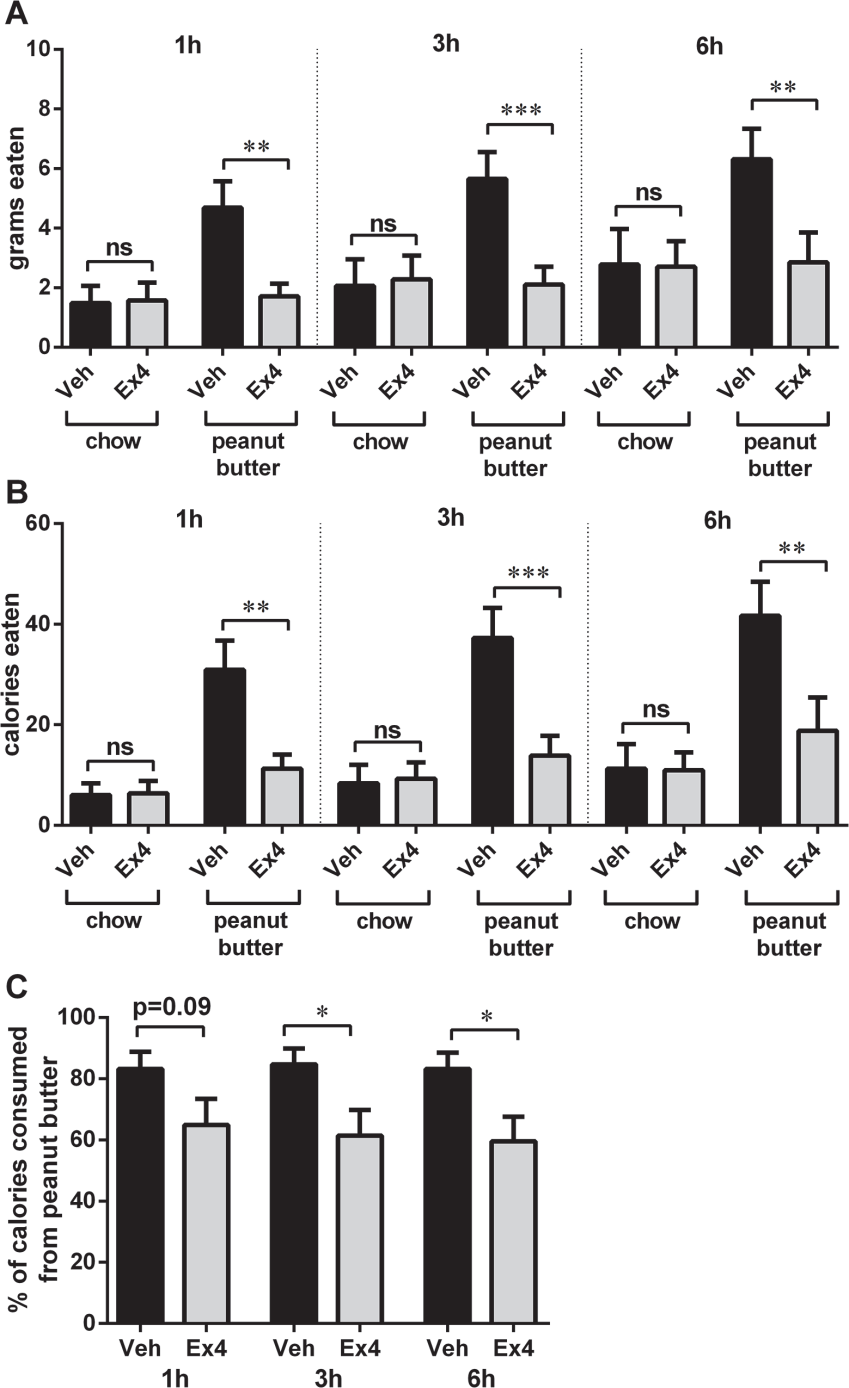
NTS GLP-1R activation preferentially affects intake of palatable food but not chow. Direct NTS GLP-1R stimulation with Ex4 suppressed the intake of palatable food (peanut butter) but not chow when both were offered simultaneously. The effect of Ex4 had a short latency (noted 1h after injection), and lasted throughout the 6h of measurements. The food intake data are represented as grams eaten (A), as calories consumed, since the two foods differ in their caloric density (B), and as the fraction of total intake (by calories) that was represented by the palatable peanut butter intake(C). Data are expressed as mean ±SEM. n = 12 per each treatment group. * p<0.05, ** p<0.01, *** p<0.005.

Direct NTS GLP-1R stimulation with Ex4 suppressed the intake of palatable food (peanut butter) but not chow *when both were offered simultaneously* (one-way ANOVAs, 1h: F_(3,33)_ = 8.52, p<0.005; 3h: F_(3,33)_ = 7.40, p<0.01; 6h: F_(3,33)_ = 4.42, p<0.05; Fig. 2). The effect of Ex4 was noted at the first, 1h, measurement time point and lasted throughout 6h of measurements. Two-way ANOVAs revealed a significant interaction between the drug treatment and food type at each time point tested (1h: F _(1, 11)_ = 6.469, p<0.05, 3h: F _(1, 11)_ = 8.163, p<0.05, 6h: F _(1, 11)_ = 7.729, p<0.05). Chow intake of vehicle-treated rats comprised 30% of their total intake while 50% of the Ex4 treated animals intake consisted of chow (with respect to amount of grams consumed during the experiment), thus while the intake of chow in vehicle-treated rats was lower than that of peanut butter, it was not negligible. Total amount of calories eaten (chow and peanut butter combined) was as follows: 1h intake vehicle: 37.0±5.9 Ex4:17.7±4.9 p<0.0005; 3h intake vehicle: 45.7±7.3, Ex4:23.2±6.7 p<0.005; 6h intake vehicle: 53.0±9.1, Ex4:29.8±9.3 p<0.05. Three rats that were determined to have cannula placements not reaching the NTS (injections were localized mostly to the ventral edge of the cerebellum) did not significantly reduce the palatable food or chow intake after Ex4 treatment (vehicle: 9.9±4.2, Ex4: 6.9±2.2 and vehicle: 5.4±2.0, Ex4: 5.3±1.6 for chow and peanut butter respectively; one-way ANOVAs, 6h: F_(3,9)_ = 1.1, p = 0.4).

### Intake of chow when offered alone

**Fig 3:**
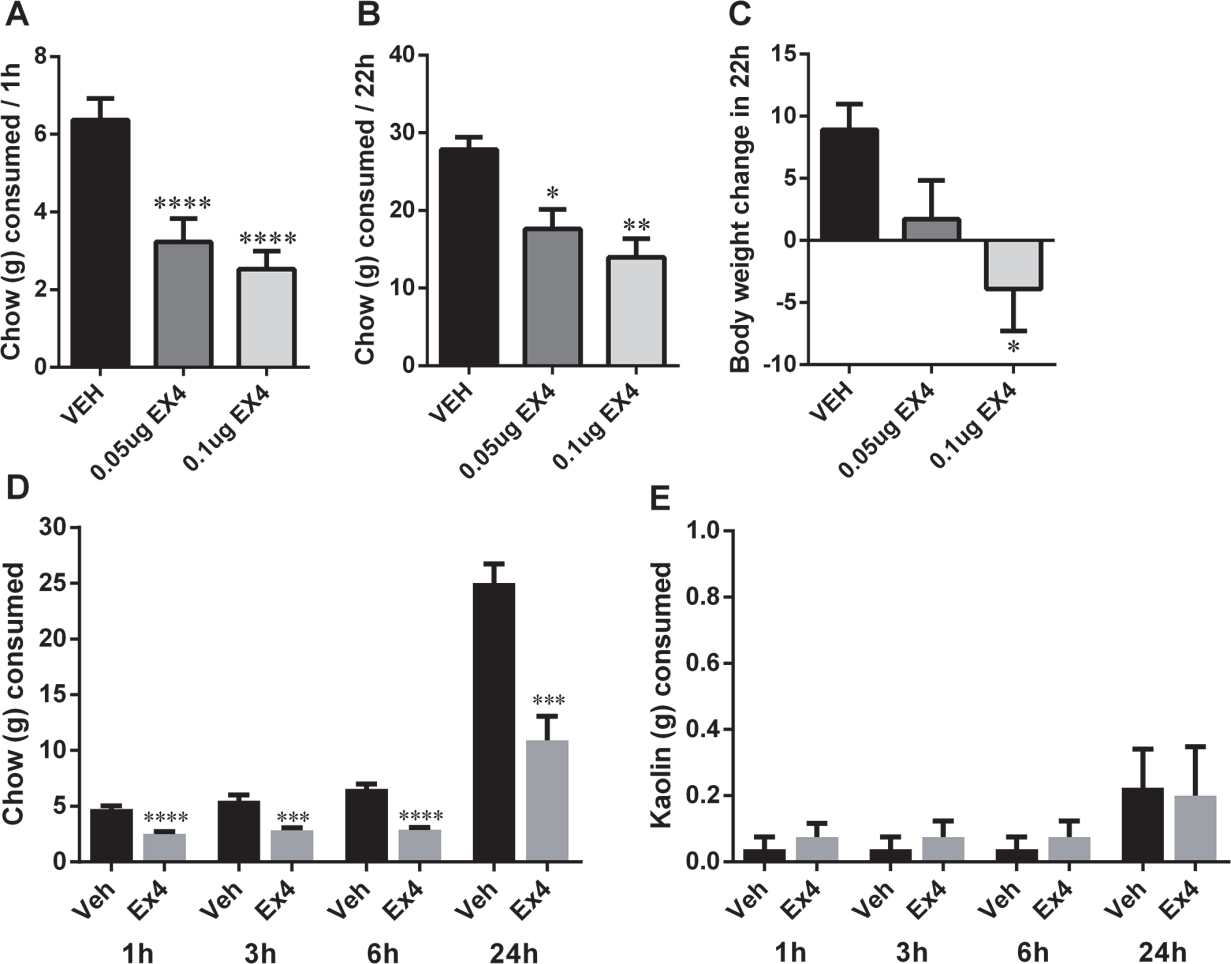
GLP-1R stimulation by Ex4 in the NTS reduces chow intake and body weight. Intra-NTS delivery of Ex4 reduced the consumption of chow over the 22h period of data collection (A-B). Body weight (g) was also reduced 22h after injections (C). In a second group of rats intake of kaolin (PICA response) was measured simultaneously with chow intake. While the chow intake was significantly reduced after intra NTS Ex4 administration (D), intake of kaolin was not altered by Ex4 (E). Data are expressed as mean ±SEM. n = 11 per each treatment group (A-C), n = 8 per each treatment group (D-E). * p<0.05, ** p<0.01, ***P < 0.005, **** p<0.0005.

However, *when chow was offered as the sole source of food*, *intake of chow was significantly reduced* after intra-NTS Ex4 application (Fig. 3). In this experimental setup the chow intake at 1h (one-way ANOVA: F_(2,20)_ = 24.12, p<0.0001; Fig. 3A) and 22h (one-way ANOVA: F_(2,20)_ = 9.56, p<0.005; Fig. 3B) was significantly reduced by both doses of Ex4. The body weight measured at 22h was also significantly reduced after the NTS Ex4 microinjection (one-way ANOVA: F(_2,20_) = 4.11, p<0.05; Fig. 3C).

### PICA

Intra-NTS administered Ex4 did not induce a PICA response at a dose of Ex4 that potently reduced 1,3,6, and 24h chow intake (nearly a 50% reduction; Fig. 3D). The lack of PICA response, as measured by lack of significant increase in kaolin intake at any time point measured (Fig. 3E), may indicate that intra-NTS GLP-1R activation, at least at an Ex4 dose of 0.05 μg, is not associated with nausea.

### NTS GLP-1R activation decreased food-motivated behavior

**Fig 4:**
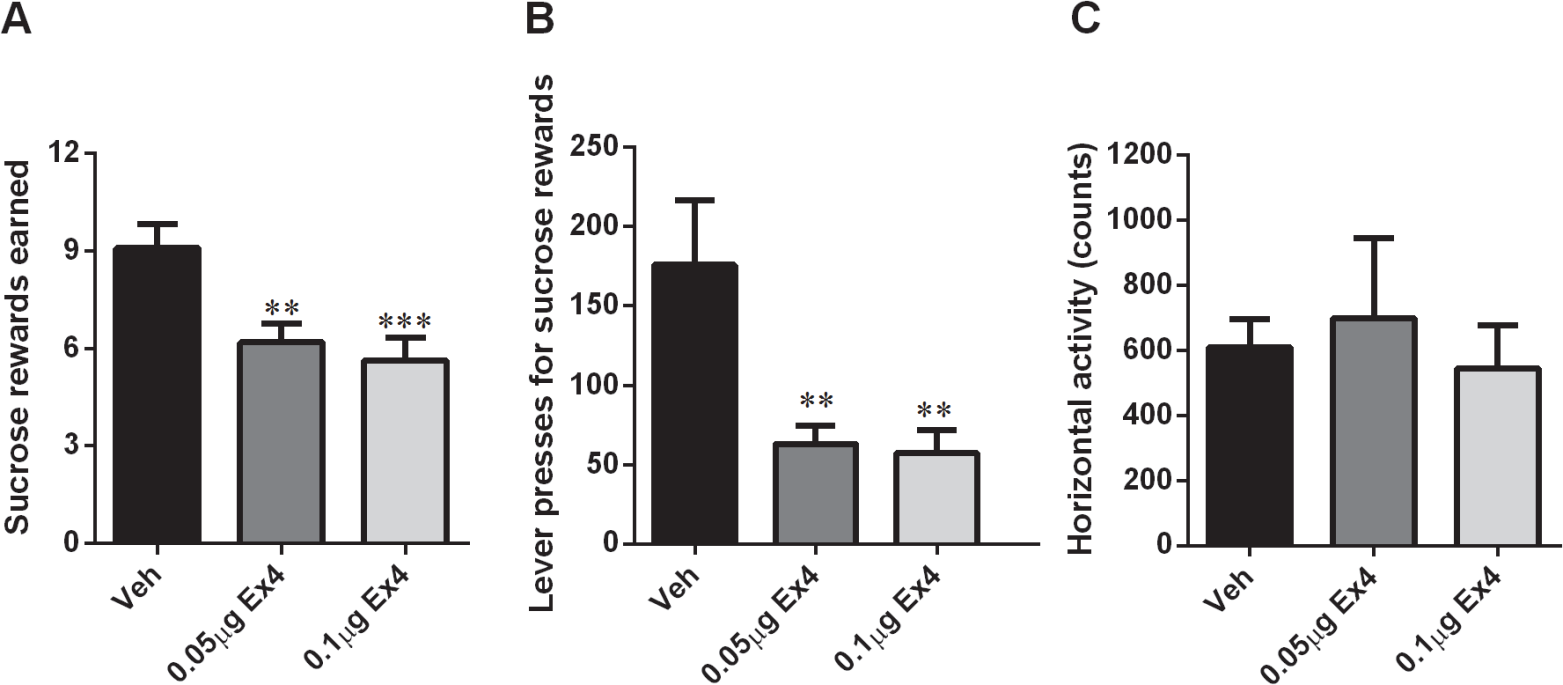
GLP-1R stimulation in the NTS decreased food-motivated behavior. The effect of intra-NTS injection of Ex4 on progressive ratio operant responding for sucrose was tested. Ex4 potently decreased the number of sucrose rewards earned (A) and the number of active lever presses (B) in an operant lever-pressing paradigm. Importantly this suppression in food-motivated behavior was not associated with a reduction in locomotor activity (C). n = 11 per each treatment group. ** p<0.01, ***P < 0.0005.

Rats responding for a sucrose reward (45mg pellet) under a progressive ratio reinforcement schedule (i.e. a schedule in which the number of presses required to obtain a single sucrose pellet increases progressively) were treated with a selective, long-lasting GLP-1R agonist, Ex4 (0.05 or 0.1 μg/0.3 μl) 10 min prior to placement in the operant boxes. The doses were chosen based on [18]. Intra-NTS administered Ex4 significantly and potently decreased the number of sucrose rewards earned (one-way ANOVA: F_(2,20)_ = 12.48, p<0.0005; Fig. 4A) and the number of lever presses emitted for sucrose (one-way ANOVA: F_(2,20)_ = 9.82, p<0.005; Fig. 4B). Post-hoc Tukey tests indicated that both doses of Ex4 were effective at reducing the number of rewards earned and lever presses. These reductions in food-motivated behavior were not accompanied by a general reduction in locomotor activity (Fig. 4C).

**Fig 5:**
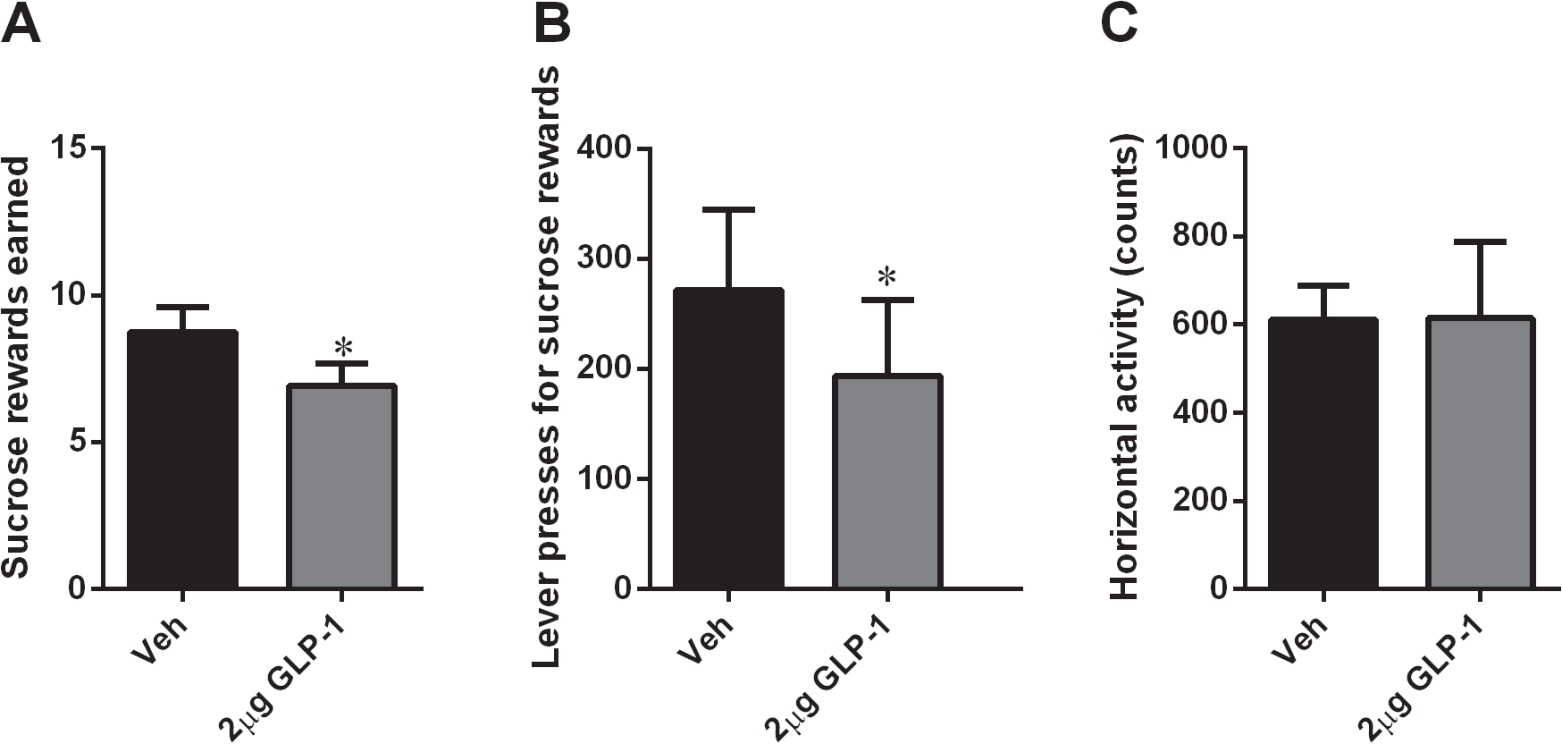
GLP-1 injected into the NTS decreased food-motivated behavior. GLP-1 decreased the number of sucrose rewards earned (A) and the number of active lever presses (B) in an operant lever-pressing paradigm, and this suppression in food-motivated behavior was not associated with a reduction in locomotor activity (C). n = 11 per each treatment group. * p<0.05.

Similar results were obtained after intra-NTS application of the endogenous peptide: GLP-1 (2.0 μg/0.3 μl), though the effect of GLP-1 was much less potent compared to that of Ex4. The number of sucrose rewards earned (p<0.05; Fig. 5A) and the number of lever presses emitted for sucrose (p<0.05; Fig. 5B) were both significantly reduced after GLP-1 treatment. These reductions in food-motivated behavior were not accompanied by a general reduction in locomotor activity (Fig. 5C).

### NTS GLP-1R activation decreased food-reward behavior

**Fig 6:**
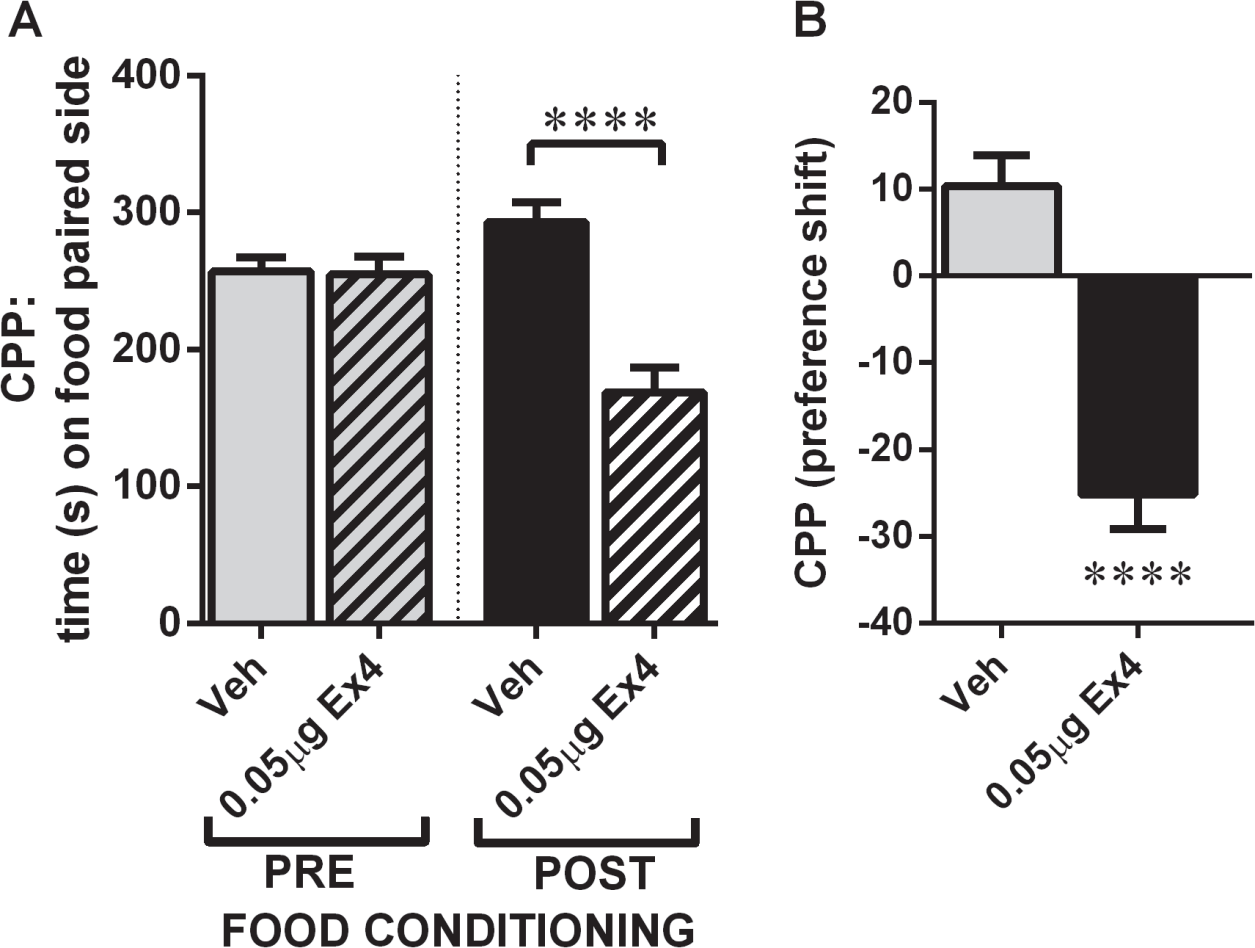
GLP-1R stimulation in the NTS decreased food reward behavior. The effect of intra-NTS injection of Ex4 on the ability of palatable food to condition a place preference was tested. Preference for the chamber paired to palatable food was abolished by Ex4 treatment. The preference [% conditioned place preference (CPP)] was calculated using the following formula: ((test − pre-test)/(total time − pre-test)) × 100. n = 11 (vehicle group) and n = 8 (Ex4 group). ****p < 0.0005. Data represent mean ±SEM.

The food-induced CPP is a complementary test of food reward behavior, in which a rat shows preference for a chamber previously paired with palatable food over a chamber previously paired with no food exposure. The CPP test informs how rewarding the rat finds the palatable food. Importantly, during the CPP test rats do not have access to food enabling dissociation of the intake of palatable food from the reward evaluation process. Rats microinjected with Ex4 (0.05 μg) into the NTS 10 min prior to the CPP test spent significantly less time in the food-paired chamber compared to the vehicle-treated group, and unlike the vehicle treated rats, showed a preference for the non-food paired chamber (Fig. 6).

### NTS expression of CCK and two enzymes key in noradrenaline synthesis after central GLP-1R stimulation

**Fig 7:**
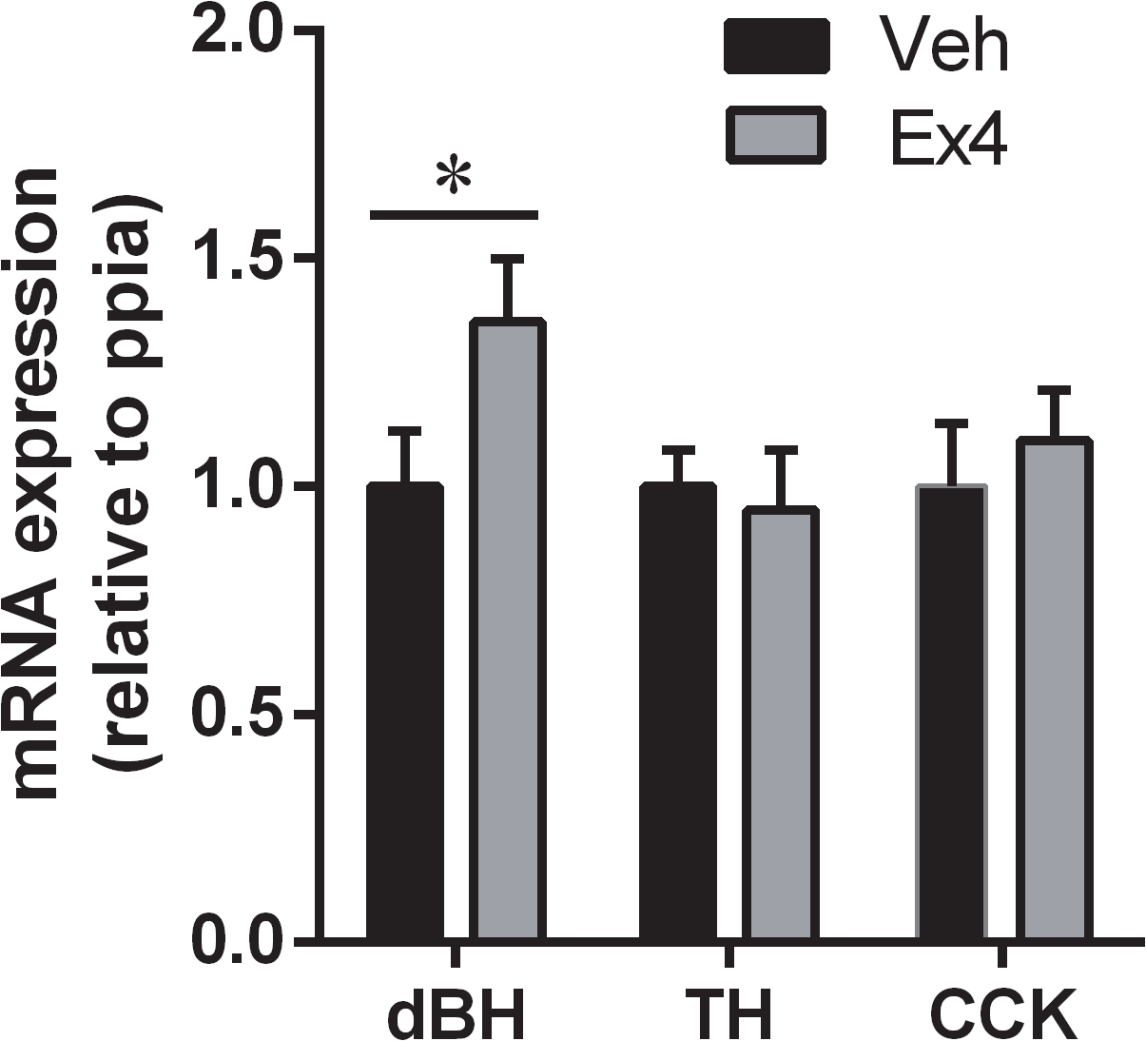
NTS expression of two enzymes key in noradrenaline synthesis after central GLP-1R stimulation. Central activation of GLP-1R with Ex4 increases the expression of dBH but not TH or CCK. Data are expressed as mean ±SEM. n = 9 (pair-fed control group), n = 11 (*ad libitum* fed control group) and n = 10 (Ex4 group). *p<0.05. Tyrosine hydroxylase (*TH*) and dopamine-beta-hydroxylase (*dBH*).

Central activation of the GLP-1R with Ex4 increased the expression of dBH (Fig. 7), an enzyme that catalyzes the hydroxylation of dopamine to noradrenaline. The expression of TH, an enzyme that catalyzes an earlier step in the noradrenaline synthesis (the reaction in which L-tyrosine is hydroxylated to obtain L-DOPA) was not altered by Ex4 treatment. The expression of CCK was also unaltered.

### GLP-1 fibers closely appose NTS noradrenergic neurons

**Fig 8:**
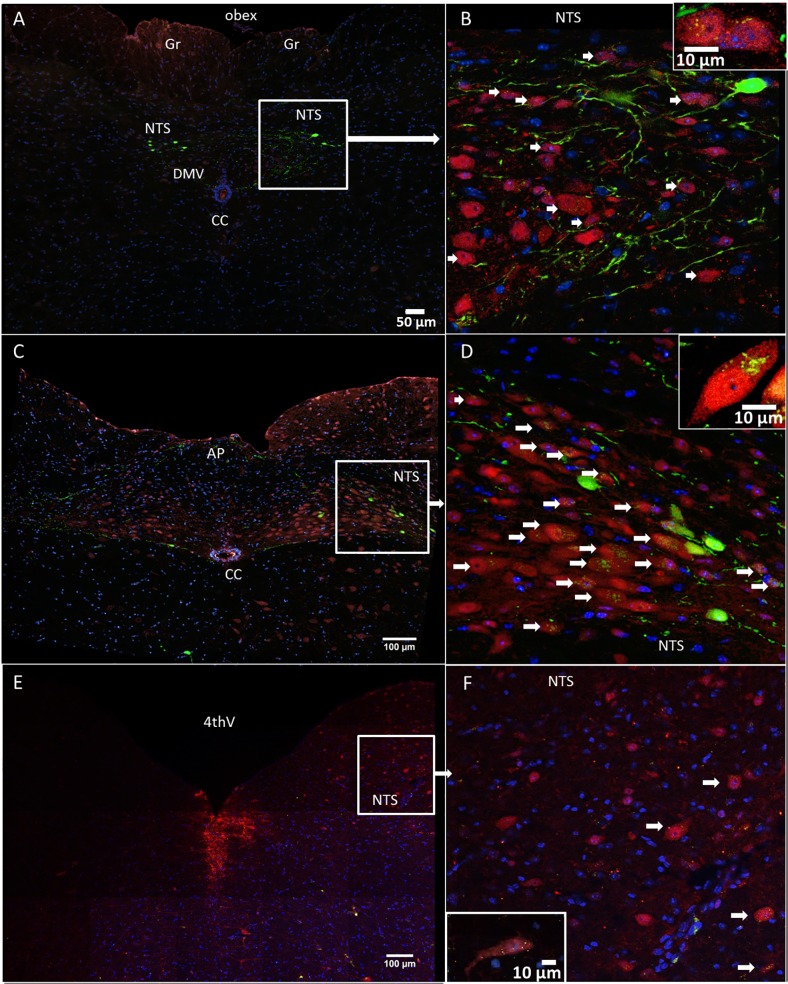
Many YFP-immunoreactive axons (green) closely apposed the TH-positive neurons (red) of the NTS. Fluorescent YFP– preproglucagon neurons (green) and DAPI (nuclear stain, blue) in coronal sections through the NTS of YFP–PPG mice. Micrographs showing the caudal NTS (A-B), the NTS at the level of the area postrema (C-D) and the NTS at the level of the 4^th^ ventricle (E-F). Cell bodies of YFP-immunoreactive preproglucagon neurons (green) were detected at the level of the area postrema and just caudally to the area postrema (A-D). Many green YFP-immunoreactive axons closely appose blue DAPI-labeled cell bodies in the NTS. White arrows indicate NTS TH-positive neuronal cell bodies closely apposed by the GLP-1 fibers. Insets in panels B,D and F show the interaction at a single neuron level. Area postrema (AP), central canal (cc), dorsal motor nucleus of the vagus (DMV), gracile nucleus (Gr), 4^th^ ventricle (4thV). B,D and F show higher magnification of areas in A,C and D, respectively.

Guided by previous data showing that neurons expressing the enzyme TH (a marker for catecholamine neurons) in the NTS project to both key mesolimbic nuclei [22,29], the VTA and the nucleus accumbens, we set out to determine whether GLP-1 fibers can be found in close apposition to the NTS TH-positive neurons. TH-immunoreactive neurons that received close appositions from green YFP-positive fibers were found at several levels of the NTS. The TH-positive neurons were most prevalent at the level of the area postrema and just caudally from area postrema (Fig. 8 A-D); at the level of the caudal 4^th^ ventricle the TH-positive neurons were more sparse (Fig. 8 E-F). The distribution of the YFP-labeled cell bodies (Fig. 8 A-D), detected within the caudal lateral NTS and lateral NTS at the level of the area postrema, was consistent with previous literature [11,12]. Few GLP-1-producing cell bodies were also found near the hypoglossal nucleus (Fig. 8C) [14]. Consistent with previous reports [12,29,30], we did not see any colocalization between TH-positive and YFP-labeled cell bodies, which indicated that GLP-1-producing neurons are likely not producing noradrenaline. It should be noted that TH-immunoreactivity within the NTS, particularly in the rostral sections of this nucleus, may also be found in C2 adrenergic neurons in addition to A2 adrenergic neurons. However, since C2 neurons are relatively sparse in the NTS at the level of the area postrema and just caudal to this area, most TH-immunoreactive neurons in this area are noradrenergic, thus allowing the conclusion that GLP-1 fibers closely appose noradrenergic neurons in the NTS.

### Mesolimbic gene expression

**Fig 9:**
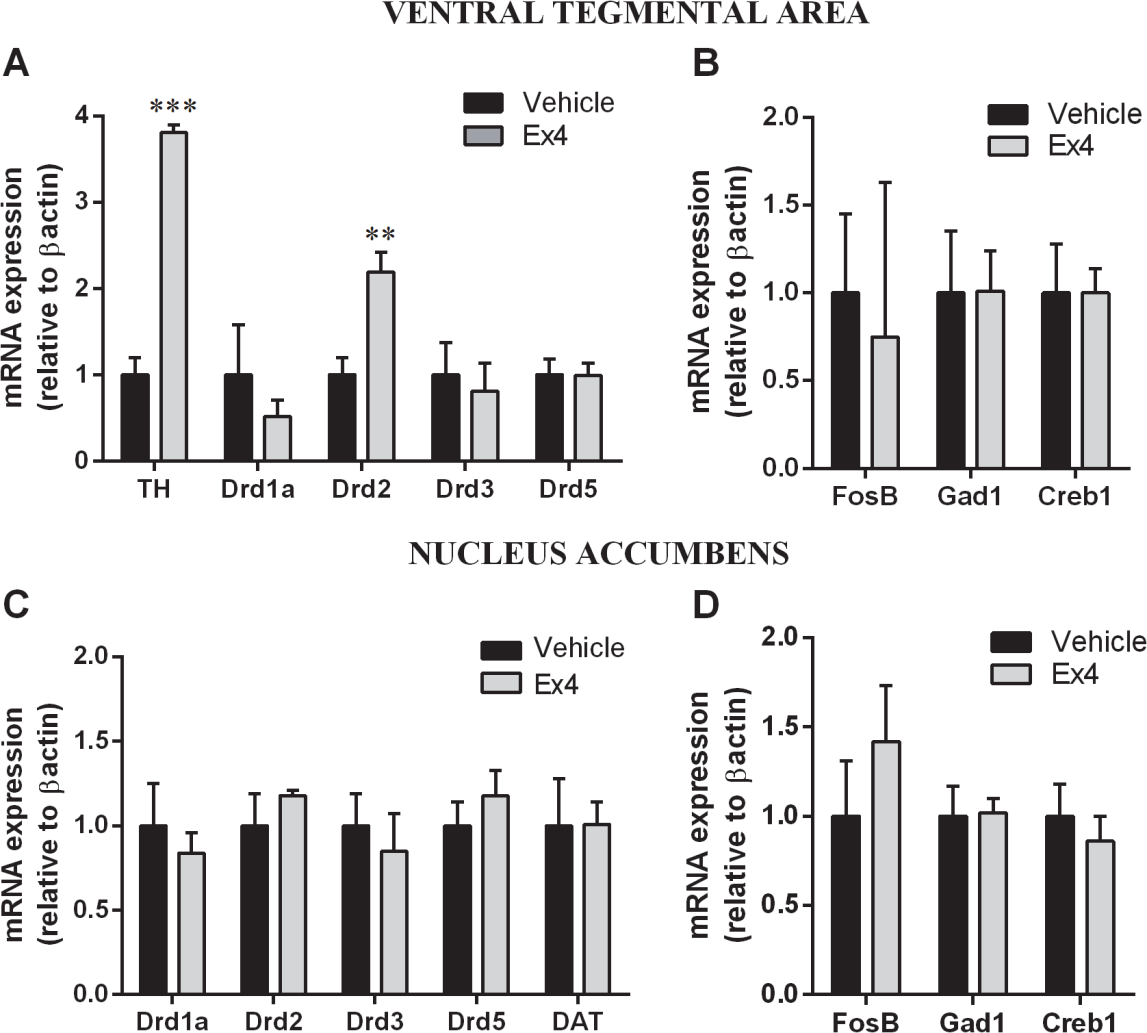
Activation of GLP-1R in the NTS alters gene expression in the mesolimbic reward system. GLP-1R activation by Ex4 in the NTS increased the mRNA expression of the gene that encodes tyrosine hydroxylase (*TH*), and dopamine 2 receptor (*Drd2*) without significantly changing the mRNA expression of other dopamine receptors in the VTA (A). The expression of several other genes previously associated with changes in reward behavior: *FosB*, *Creb1* and *Gad1* remained unchanged after intra-NTS Ex4 treatment (B). Intra-NTS Ex4 treatment did not alter the expression of dopamine receptors, dopamine transporter (DAT), *FosB*, *Gad1* or *Creb1* in the nucleus accumbens (Fig C-D). Data are expressed as mean ±SEM. n = 6 (vehicle group) and n = 5 (Ex4 group). ** p<0.01, *** p<0.005. Dopamine receptor 1 (*Drd1a*), dopamine receptor 3 (*Drd3*), dopamine receptor 5 (*Drd5*), glutamate decarboxylase 1 (*Gad1*), cAMP responsive element binding protein 1 (*Creb1*), FBJ osteosarcoma viral oncogene B (*FosB*).

GLP-1R stimulation in the NTS resulted in a nearly fourfold increase in expression of mRNA encoding TH (Fig. 9A), an enzyme required for the synthesis of dopamine in the VTA and a marker for dopamine neurons in this area. Furthermore, the expression of dopamine 2 receptor (D2R) in the VTA was twofold increased after intra-NTS Ex4 microinjection (Fig. 9A). The expression of other dopamine receptors was not altered (Fig. 9A). Similarly the expression of several other genes previously associated with changes in reward behavior: *FosB*, transcription factor (*Creb1*), as well as the gene encoding glutamate decarboxylase (*Gad1*) remained unchanged after intra-NTS Ex4 treatment in this experimental paradigm (Fig. 9B). Intra-NTS Ex4 treatment did not alter the expression of dopamine receptors, dopamine transporter (DAT), *FosB*, *Gad1* or *Creb1* in the nucleus accumbens (Fig. 9C-D).

## Discussion

GLP-1, through its activity on the central receptors, is a key regulator of food intake [8–10,31]. Recently it was also suggested that GLP-1 is essential for control of food reward behavior [18]. Much of the earlier literature reports are focused on neuroanatomical substrates associated with either homeostatic or hedonic feeding. For GLP-1, the hypothalamus and the hindbrain are considered key sites from which GLP-1 reduces ingestive behavior, while reward effects of GLP-1 are ascribed to the mesolimbic GLP-1R [18,32,33]. Here we report six findings that provide key information about the impact of activation of GLP-1R in the hindbrain, specifically the NTS, on reward behavior: 1) NTS-directed GLP-1R activation resulted in a selective reduction in intake of palatable food. 2) Both GLP-1 and Ex4 microinjected into the NTS suppressed food-motivated behavior for sucrose in a progressive ratio test. 3) NTS GLP-1R activation suppressed food reward behavior in a CPP test. 4) NTS GLP-1-producing neurons are found in close apposition to the NTS noradrenergic neurons, previously shown to send direct projections to the mesolimbic system, specifically the nucleus accumbens and the VTA, providing a potential direct neuroanatomical link between GLP-1 action in the NTS and the mesolimbic system. 5) Central GLP-1R stimulation also increases the NTS expression of dBH, key enzyme in noradrenaline synthesis providing a biological link between GLP-1R activation and NTS noradrenergic neurons. 6) NTS GLP-1R activation altered the expression of dopamine-related genes in the VTA.

Rats given a choice of consuming highly palatable peanut butter or less palatable chow chose to consume nearly four times more calories from the palatable food compared to chow. However, the NTS-directed activation of the GLP-1R attenuated this preference by selectively reducing the amount of palatable food consumed. Thus, it seems that this GLP-1R manipulation selectively affected intake of palatable food. The role for the caudal brainstem, specifically the area postrema and the NTS, in the palatable food-selective ingestive behavior control, was suggested by previous studies utilizing chemical lesions of these regions. Rats with capsaicin-induced neuronal damage to the area postrema and NTS regions overconsumed palatable food, but not chow [34,35]. These rats did not exhibit any other behavioral or physiological changes, underscoring the specificity of this effect. These studies concluded that the capsaicin lesions selectively impair a discrete population of neurons that normally inhibits the exaggerated consumption of preferred food. In light of the current data, an interesting possibility could be that the inputs to the NTS GLP-1-producing neurons, or their downstream neuronal targets within the NTS, may represent one of these discrete populations, inputs to which are disrupted by the hindbrain-injected capsaicin resulting in an exaggerated intake of palatable preferred foods.

The palatable food selective ingestive changes seen here after intra-NTS GLP-1R activation are also akin to those already demonstrated for central blockade of opioid receptors [for review see: [36,37]]. Similarly to the current results, in a no-choice paradigm (animals are offered only one type of food at a time), opioids change the intake of any kind of food, however when the animals are given a choice opioids selectively alter the intakes of preferred foods [38–40]. There is a neuroanatomical overlap between the opioid and the GLP-1 control of feeding; NTS-selective mu-opioid receptor stimulation and blockade were shown to increase and decrease food intake respectively [41]. The potential functional interaction of the two systems in the hindbrain to control intake of preferred foods is an interesting emerging hypothesis for future investigation. The differential effect of Ex4 on chow intake in a choice vs non-choice food test was already demonstrated in the mesolimbic system, via microinjection of Ex4 into the VTA and the nucleus accumbens shell, where concurrent offering of high-fat and chow resulted in a selective reduction of the high-fat intake [42]. However, when chow was offered alone a potent reduction of chow intake was observed [18]. Since the rats choose to consume more of the palatable food than chow, an alternative explanation to the reduced reward hypothesis could be that Ex4 simply reduces the intake of the principle source of calories. Thus, the food choice experiment alone would not be sufficient to conclude that NTS GLP-1R activation reduces food reward. However, considering that both progressive ratio operant conditioning and CPP for food were reduced by Ex4, both more direct tests of food reward, we conclude that Ex4 does reduce food reward behavior when injected into the NTS. Additionally, in the current study rats consuming chow alone were food restricted to 50% of their regular overnight food intake. Food restriction increases the palatability of any available source of calories, making the consumption of any available food rewarding. Thus, while we cannot eliminate the possibility that mechanisms other than reward suppression are involved in the chow intake suppression after NTS GLP-1R activation, due to the background of food restriction, food reward suppression represents one potential mechanism of reduced chow consumption in the current study.

Two complementary tests of food reward behavior used here, progressive ratio operant conditioning and CPP, clearly demonstrate a role for NTS GLP-1R in food reward suppression. The intra-NTS Ex4 microinjection potently reduced the rats’ motivation to work for sucrose pellets. These rats earned 30% less sucrose reward and were willing to put in only a third of the effort (active lever presses) compared to the vehicle-injected control rats. Also in the CPP test, a test that allows complete separation of food consumption and food reward seeking (since no food is offered on the testing day), the intra-NTS Ex4 injected rats showed a suppressed food reward behavior. Unlike control rats, which showed a small but significant increase in the amount of time spent in the environment paired with palatable food during training, the Ex4-treated rats chose to spend significantly more time in the non-food paired environment indicating reduced food-reward seeking behavior. Notably, the changes in reward behavior demonstrated here were not associated with general locomotor inhibition. These results are in line with a recent report that demonstrates that NTS Ex4 can reduce food-reward behavior even 3h after injection [43]. We also report here, for the first time, that the endogenous peptide GLP-1 reduces food reward behavior in the progressive ratio operant task. This is an important finding that strengthens the overall idea of the reward-suppressing role of the central GLP-1 system and attends to the potential criticism posing that the reward-suppressing action of Ex4 is drug-specific. The idea of a differential action of Ex4 and GLP-1 in the brain was suggested by previous studies [44].

We have previously showed that VTA and nucleus accumbens GLP-1R activation with Ex4 reduces food reward behavior [18]. These two mesolimbic sites represent classic neuroanatomical sites accepted for their role in regulation of reward derived from food and drugs of addiction. Interestingly our current results, combined with the previously reported intra-mesolimbic Ex4 injections, indicate that the NTS GLP-1R were just as sensitive to the reward suppressing action of Ex4 as their VTA and nucleus accumbens counterparts. Thus, even though the caudal brainstem in general, and NTS in particular, is rarely thought of as a reward modulating CNS region, current results clearly call for its addition to the reward-control brain sites.

The mesolimbic circuitry plays a key role in the control of reward behavior. Thus, it is likely that in order for the hindbrain GLP-1R activation to affect reward behavior the NTS GLP-1R-induced activity should be ultimately transmitted to the mesolimbic system. NTS neurons expressing the enzyme TH (a potential label for noradrenergic and adrenergic neurons in the NTS) were previously shown to send direct projections to the nucleus accumbens and the VTA [22,29,45]. In fact NTS A2 neurons are a major source of noradrenaline to the nucleus accumbens and these A2 neurons were already implicated to be crucial in opioid reward and withdrawal behavior [46,47]. Current data indicate that the NTS TH-positive neurons are closely apposed by GLP-1 fibers. Importantly the location of the GLP-1-fiber apposed TH-cell bodies is in line with that previously indicated for nucleus accumbens-projecting TH-positive neurons [22]. These results are in line with one previous study [30]. This neuroanatomical evidence suggests that GLP-1 and activation of GLP-1R in the NTS may reach the key mesolimbic nuclei by changing the activity of the TH-positive neurons. This idea is further supported by our data showing that central GLP-1R activation with Ex4 increases the expression of a key enzyme required for noradrenaline synthesis, dBH. Other possible routes via which GLP-1R activation in the NTS could affect mesolimbic activity may include changing the activity of GLP-1, CCK or neurotensin-positive neurons since all three have been retrogradely labeled from the nucleus accumbens to the NTS [22,42]. The activation of NTS CCK neurons by GLP-1 treatment is not supported by our gene expression data. Also, the activation of GLP-1 neurons by GLP-1R stimulation in the NTS seems unlikely since, at least in a mouse, GLP-1 neurons do not express GLP-1R [48].

Further evidence for the NTS GLP-1R stimulation impacting on activity in the mesolimbic system is provided by current data showing that dopamine-related gene expression in the VTA is altered by NTS GLP-1R stimulation. We show that dopamine 2 receptor (D2R) expression was selectively twofold increased by GLP-1R activation in the NTS, leaving the expression of dopamine 1, 3 and 5 receptor genes unchanged. In the VTA D2R are primarily thought to function as autoreceptors that decrease the activity of the dopaminergic neurons they are expressed by. Interestingly only select populations of dopaminergic neurons may be under this inhibitory control of D2R [49]. Specifically, the majority of the dopaminergic neurons that innervate the nucleus accumbens are inhibited by D2R activation, however nearly none of the dopamine neurons projecting to the amygdala are sensitive to this inhibition [49]. Thus the upregulation of D2R reported here may be consistent with a reduction of the ability of accumbens-projecting dopamine neurons to be activated by reward signals like the palatable food used here. This idea is consistent with the suppression of food-reward behavior. The expression of TH, an enzyme in the VTA associated with an increased production of dopamine, was nearly fourfold increased by the NTS-directed Ex4 treatment. One previous study reported a similar increase in TH protein levels in the VTA after intra-VTA GLP-1R activation [50], thus both GLP-1R populations, in the VTA and in the NTS, despite their neuroanatomical distance, may exert a similar effect on the VTA dopamine production. The projection target/s of the dopaminergic neurons that increase their TH production in response to NTS GLP-1R activation remains to be determined. However, based on previous data it is likely that it is the amygdala. Central GLP-1R activation via lateral ventricle injection of GLP-1R agonist, likely reaching many GLP-1R populations in the brain including those in the VTA and NTS, increases dopamine release in the amygdala [51]. Increased dopamine activity in the amygdala was associated with reduced food-motivated behavior for sucrose [51]. The case for the amygdala as a likely target may also be strengthened by the fact that amygdala-projecting dopamine neurons would not be inhibited by the simultaneous increase in D2R-mediated inhibition. Alternatively (or in addition to the amygdala release) the increased TH levels in the VTA may contribute to increased somatodendritic release of dopamine in the VTA [52], associated with inhibition of nucleus accumbens and cortex-projecting dopamine neurons.

The reward suppressing GLP-1-driven circuitry outlined here may be engaged by both central and peripheral energy balance controlling signals. Leptin, released from the adipose tissue, has been shown to activate GLP-1 neurons. The anorexic effect of leptin may be partly mediated by GLP-1 [53]. However, just as hindbrain GLP-1R activation may be participating in food reward control, the hindbrain action of leptin may also extend to reward behavior control. Supportively, recent data indicate that activation of leptin receptors in the medial NTS results in a selective reduction in reward derived from food, but not drugs of abuse like morphine [19]. Considering that both hindbrain GLP-1 and leptin receptor activation leads to reward suppression, and the previous studies already show a functional interaction between the two systems. It is possible that the food reward-suppressing effects of leptin are mediated by the central GLP-1 system.

In addition to peripheral signals, NTS neurons may also be engaged by central signals descending from the forebrain in order to regulate food reward behavior. For example orexin, an orexigenic neuropeptide produced in the lateral hypothalamus previously shown to increase food reward behavior [54,55], may be released in the NTS [56,57] to increase the rewarding value of food and food-motivated behavior [20]. Furthermore orexin neurons may innervate the NTS GLP-1 neurons as well as the NTS TH neurons [56]. Thus the two systems, GLP-1 and orexin, may interact within the NTS both by acting on the same downstream neurons (possibly the noradrenergic neurons already shown to link the NTS to the mesolimbic reward system), or alternatively orexin may directly inhibit the GLP-1-producing neurons.

The central GLP-1 system is engaged by signals associated with viscerosensory malaise and formation of consequent conditioned taste aversions, conditions that are associated with reduced food intake. Hindbrain administration of GLP-1 agonists is not associated with conditioned taste aversions [58]. However, medial NTS administration of Ex4 has previously been shown to induce PICA, consumption of non-nutritive substances that may be indicative of viscerosensory malaise [58]. However, the feeding suppression induced by NTS GLP-1R activation does not seem to rely on nausea induction, since at low doses of NTS Ex4, that still potently reduce food intake and reward, our current data and others[43] show that Ex4 does not induce the PICA response. Lack of a sickness response is also supported by the fact that there are no changes in locomotor activity, as sickness responses are typically associated with a reduction in motor activity. Collectively, while it is clear that feeding suppression resulting from NTS GLP-1 stimulation is not always accompanied by nausea, it is entirely possible that with a stronger activation of GLP-1 neurons the NTS GLP-1R activation contributes to reward behavior reduction accompanying visceral malaise. Moreover, NTS GLP-1 neurons are also a key element of the anorexia-inducing neurocircuitry that follows bacterial infection [59]. In fact, NTS noradrenergic neurons are also activated by the bacterial infection mimicking agent- lipopolysaccharide [60]. Thus the NTS GLP-1 to TH circuit suggested by current data may participate in infection-induced anorexia and food-motivated behavior suppression.

Considering the proximity of the NTS to the area postrema with its leaky blood brain barrier it is likely that peripherally injected long-acting GLP-1 analogues utilized clinically (for example Ex4 used here, or liraglutide) may also gain access to the relevant GLP-1R populations to reduce food reward behavior. Thus, the neuronal circuitry identified here may be clinically relevant. Collectively our findings support the idea that the caudal brainstem participates in the control of food reward and food motivated behavior and identify potential mechanisms via which the NTS GLP-1R-evoked signal may be transmitted to the mesolimbic pathways to reduce food reward behavior.
